# Prostate cancer malignancy detection and localization from mpMRI using auto-deep learning as one step closer to clinical utilization

**DOI:** 10.1038/s41598-022-27007-y

**Published:** 2022-12-27

**Authors:** Weiwei Zong, Eric Carver, Simeng Zhu, Eric Schaff, Daniel Chapman, Joon Lee, Hassan Bagher-Ebadian, Benjamin Movsas, Winston Wen, Tarik Alafif, Xiangyun Zong

**Affiliations:** 1 WeCare.WeTeach, Troy, MI 48098 USA; 2 Henry Ford Health System, Detroit, MI 48202 USA; 3 Trinity Health, Minot, ND 58701 USA; 4 Department of Radiology, Michigan State University, East Lansing, MI 48824 USA; 5 Department of Osteophytic, Michigan State University, East Lansing, MI 48824 USA; 6 Department of Physics, Oakland University, Rochester, MI 48309 USA; 7 SJTU-Ruijing-UIH Institute for Medical Imaging Technology, Shanghai, 200241 China; 8 Department of Radiology, Ruijin Hospital Shanghai Jiaotong University School of Medicine, Shanghai, 200031 China; 9 The Global Institute of Future Technology, Shanghai Jiaotong University, Shanghai, 200240 China; 10 Umm Al-Qura University, Jamoum, 25375 Saudi Arabia; 11 Shanghai JiaoTong University Affiliated Sixth People’s Hospital, Shanghai, 200233 China

**Keywords:** Prostate cancer, Biomedical engineering

## Abstract

Automatic diagnosis of malignant prostate cancer patients from mpMRI has been studied heavily in the past years. Model interpretation and domain drift have been the main road blocks for clinical utilization. As an extension from our previous work we trained on a public cohort with 201 patients and the cropped 2.5D slices of the prostate glands were used as the input, and the optimal model were searched in the model space using autoKeras. As an innovative move, peripheral zone (PZ) and central gland (CG) were trained and tested separately, the PZ detector and CG detector were demonstrated effective in highlighting the most suspicious slices out of a sequence, hopefully to greatly ease the workload for the physicians.

## Introduction

In this section, the literature in the field of diagnosis of prostate cancer (PCa) from medical images, the current state-of-the-art research of using deep learning algorithms in automating the diagnosis process and challenges in closing the gap of research and clinical utilization is discussed.

### Background

Prostate cancer (PCa) has been studied for its commonality and high mortality rate in the United States. As recommended by almost all the cancer types, early detection through regular screening plays an essential role in reducing the mortality rate and improving quality of life for PCa patients (https://www.cancer.org/cancer/prostate-cancer/about/key-statistics.html). In clinics, the diagnosis is made based on the screenings and the biopsies. To be specific, the biopsy serves as the golden standard when it comes to determining the malignancy of a suspicious lesion. However, the procedure of a typical prostate biopsy is invasive and complications such as hemorrhage, dysuria and infections have been reported. Cases of disagreement between the biopsy and the screenings have been reported though rarely^1^.

Among the screening techniques, multiparametric magnetic resonance imaging (mpMRI) has shown increasing impact in clinical decision making. Popular sequences include T2-weighted (T2W), diffusion-weighted imaging (DWI), dynamic contrast-enhanced imaging (DCE) and MR spectroscopy. According to the report, best visual of PCa lesions happens when the sequences are integrated in a parametric format^2,3^.

The definition of malignancy in the clinics correlates to Gleason score 7 (including 3 + 4) in histopathology^4^ and automatic detection of which from the mpMRI is the goal of this work. To be specific, the techniques used in automating the screen reading process are machine learning and computer vision customized in this particular tasks^5^.

For the past few years, deep learning models have made progress in making diagnosis from reading chest X-ray^6^, mammogram^7^, and mpMRI^8,9^.

However, small sample size has been one of the challenges for deep machine learning algorithms to learn a good pattern from limited data and labelling. Data acquisition and sharing is a process that is concerning policy and regulations, with patients’ privacy and medical ethics put forward. Data labelling on the patient level is labor-intensive and also restricted with the regulations. While data collection is under the way for many institutions, efforts have been made to leverage from sophisticated models trained on large-scale data sets consisting of natural images^10–12^.

### Previous work

Data used in the study was originated from the SPIE-AAPM-NCI Prostate MR Gleason Grade Group Challenge^13^, which aimed to develop quantitative mpMRI biomarkers for determination of malignant lesions in PCa patients. PCa patients were previously de-identified by SPIE-AAPM-NCI and The Cancer Imaging Archive (TCIA).

**Figure 1:**
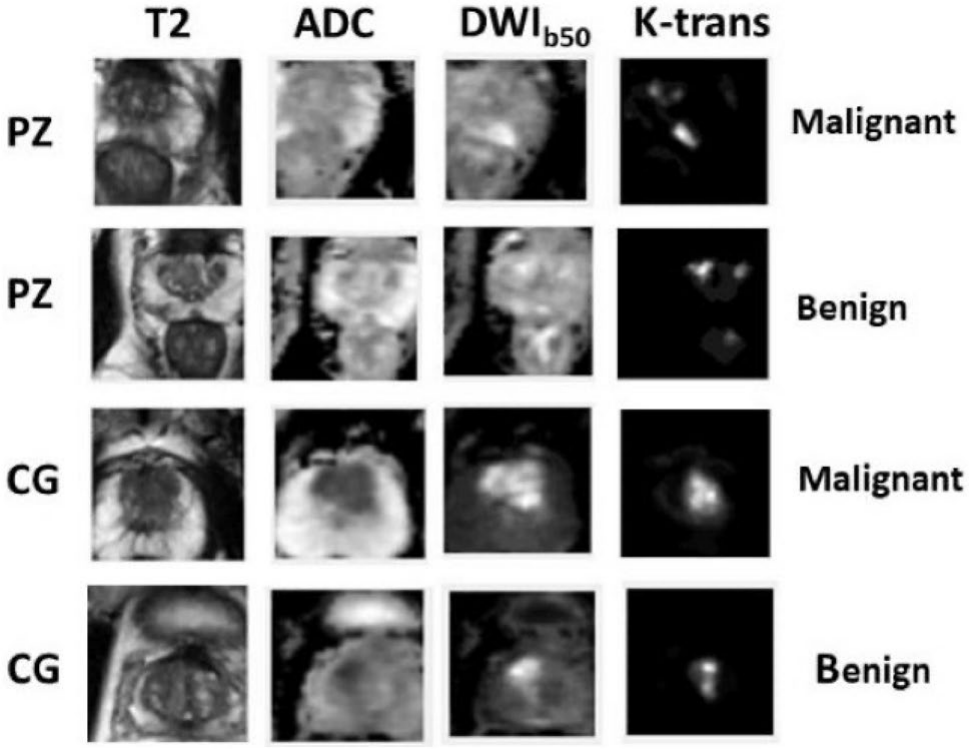
Sample images of the cropped 64 × 64 pixel rectangle from all four modalities: T2, ADC, DWI, and K-trans after resampling and registration. The lesions are malignant in PZ, benign in PZ, malignant in CG, and benign in CG from row 1 to 4 respectively.

Each patient came with four modalities displayed as in Fig. 1. Lesions exhibited hypointense signals in T2 weighted and apparent diffusion coefficient (ADC) map, and hyperintense signals in diffusion weighted imaging with low b values (b = 50). The modality of k-trans was not included in the input channels for failing to visually differentiate cancer and disease under k-trans.

In our previous work, we found that multi-modality input contributed significantly to accurate classification. In most cases, class activation map (CAM)^14^ helped provide the proof about where the model is looking at when making predictions. The central point of the potential lesion was provided^13^.

One step closer to clinical utilization by easing the workload of a medical expert is the focus and contribution of this paper. Contributions of this paper can be summarized as the following. (1) We inputted into the classification models the entire prostate gland (PZ and CG separately) rather than the cropped region of interest (ROI). (2) According to Fig. 1, lesion can exhibit very different characteristics when residing in PZ and CG. Another attempt in improving upon our previous work was to train and test using separate models for PZ and CG. (3) To verify the robustness of the trained model, we tested it on an independent cohort from our own institute . Experimental results showed that the trained PZ-detector and CG-detector were able to rank the probability of malignancy for each slice and highlight the suspicious slices out of the sequence, despite of the challenges that the testing samples are generated from different scanners with different parameters.

## Data

In this section, imaging acquisition parameters and image pre-processing steps are described in detail.

### Scanning parameters

The images were acquired on two different types of Siemens 3T MR scanners, the MAGNETOM Trio and Skyra. T2W images were acquired using a turbo spin echo sequence and had a resolution of around 0.5 mm in plane and a slice thickness of 3.6 mm. The DWI series were acquired with a single-shot echo planar imaging sequence with a resolution of 2 mm in-plane and 3.6 mm slice thickness and with diffusion-encoding gradients in three directions. Three b-values were acquired (50, 400, and 800 s/mm^2^), and subsequently, an ADC map was calculated by the scanner software.

To test cross-institutional generalization capability of our model, an independent cohort (test cohort 2) consisting of 40 patients was collected for testing from our own institution with the IRB-approval. An ultrasound-guided needle biopsy was performed to confirm the diagnosis. Two image modalities were acquired for each patient using a 3.0 T MR scanner (Ingenia, Philips Medical System, the Netherlands) using small field of view as follows: T2W acquired with Fast-Spin-Echo (TE/TR: 4389/110ms, Flip Angle: 90° with image resolution of 0.42 × 0.42 × 2.4mm^3^) and DWI with two b values (0 and 1000 s/mm^2^). The voxel-wise ADC map was constructed using these two DWIs.

### Data pre-processing

**Figure 2:**
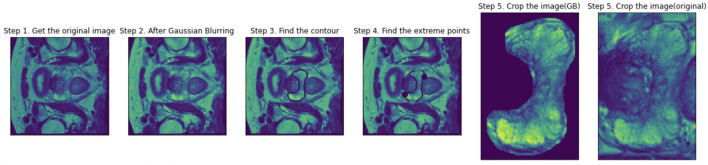
PZ cropping steps on one slice of the sequence. Gaussian blurring was applied to increase the contrast. Four extreme points were located based on the annotated contour. With a margin of 5 pixels, a surrounding rectangle was cropped. CG cropping follows similar steps.

All images were resampled and registered to T2-axial image sets in the software of MIM (https://www.mimsoftware.com). N4 bias-field correction was applied to T2 and ADC for intensity uniformity correction. Then sub-regions such as PZ or CG were cropped from the sequence based on the contouring performed by our team of medical experts. Pre-processing and cropping on one slice were shown in Fig. 2. Gaussian blurring was applied to increase the contrast. Four extreme points were located based on the annotated contour. With a margin of 5 pixels, a surrounding rectangle was cropped.

**Figure 3:**
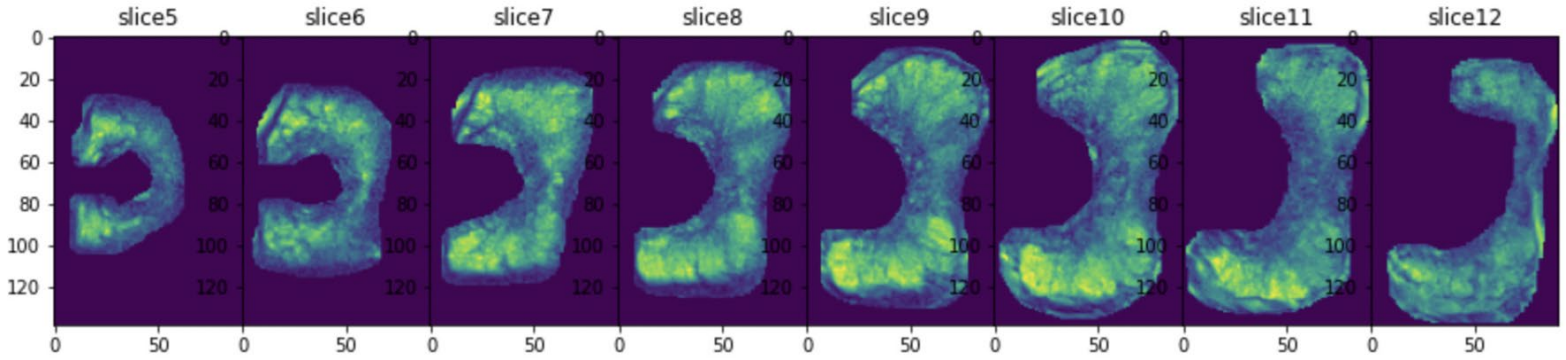
PZ cropping results on one sequence consisting of 8 slices.

**Figure 4:**
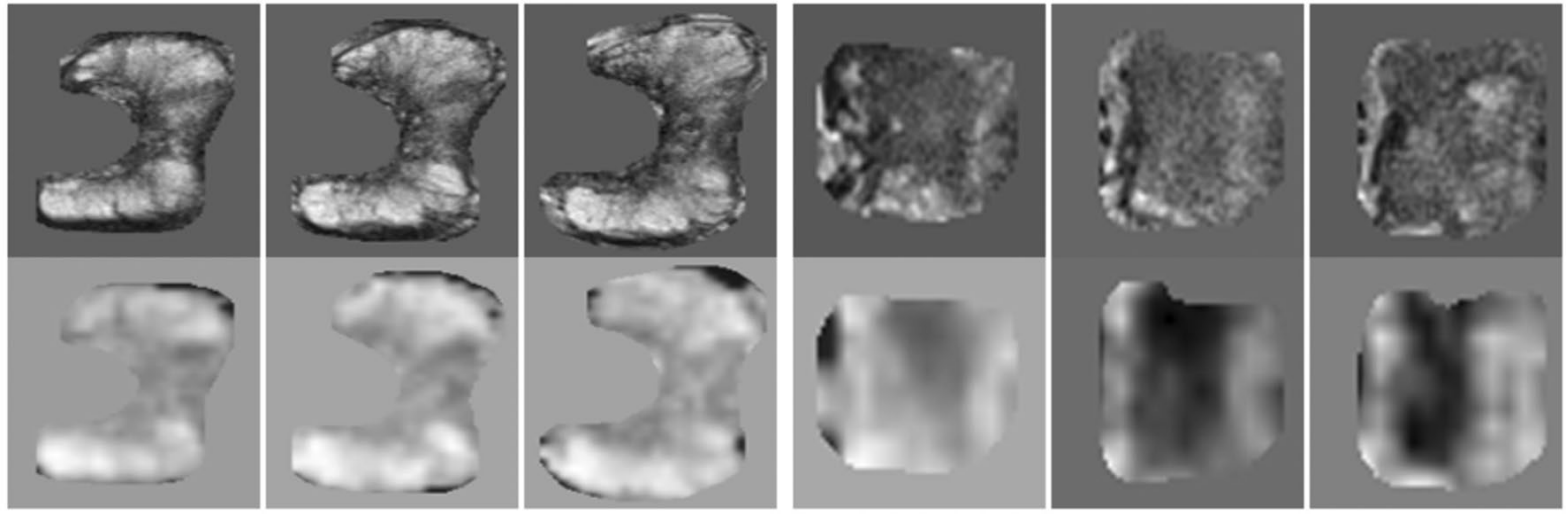
Example of cropped sub-regions with three consecutive slices. (left) PZ with Gleason Grade Group 3. (right) CG with Gleason Grade Group 2. (Upper) T2W. (Lower) ADC.

In the model training and inference stage, labelling is associated with the sequence rather than one slice. Therefore, the extreme points were searched through the sequence to not to miss any part of the PZ or CG, as shown in Figs. 3 and 4. The cropped sequence of sub-region of PZ or CG were then used as the input to train the PZ-detector and CG-detector, respectively. There are four patients with lesions spreading both PZ and CG. In these four cases, lesions were assigned to the sub-region based on the volumetric ratio from the bare eyes. In the experiment, as stated in the manuscript, we left a margin of 5 pixels when cropping the sub-region, which should leave enough room for including the lesion as an entirety.

## Method and results

### Auto-keras

Auto deep learning models for medical image analysis have not been studied much. To fully take advantage of this technique, implementation details were illustrated as follows. Codings can be found at (https://github.com/WeiweiZongHFHS).

To increase the sample size, and to leverage the 3D information between two consecutive slices, the 2-channel input was augmented to include T2-ADC pair, consecutive T2 pairs and consecutive ADC pairs. Performance of using just T2-ADC pair and the mixed pairs were compared for both PZ and CG detectors.

The number of benign lesions outnumbered malignant lesions and re-weighting was used accordingly. After shuffling the data, one third of the public data were randomly selected for validation. The trained model was then tested on the test cohort 2.

Early stopping with a patience value of 10 and optimization goal of area-under-the-curve (AUC) for validation set was used to prevent overfitting. Bayesian tuner was used as the searching strategy and maximum searching epochs were set as 20. Bayesian tuner, as opposed to the regular greedy tuner, follows an iterative process rather than searching for every possible combination of parameters. At first, a few sets of parameters were selected at random. The next set of parameters was chosen based on the performance until reaching the pre-defined performance bar or the maximum iterations. Auto-augmentation included a search in the augmentation space with operations such as random flip, translation, and contrast, etc.

**Figure 5:**
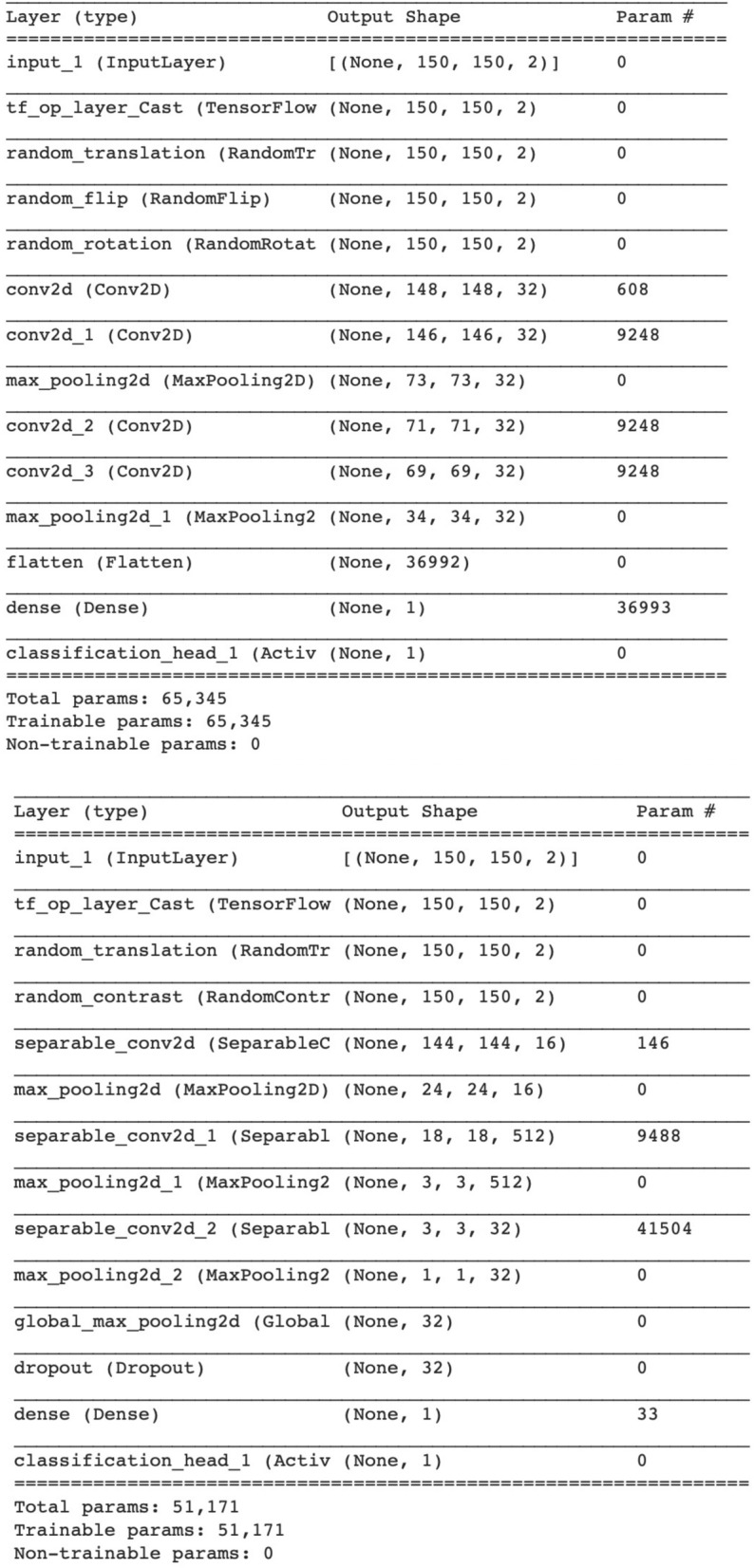
Customized models with layer type, output shape and number of parameters for each layer to detect lesions in PZ (Upper) and CG (Lower), respectively.

The learned optimal models to detect lesions in PZ and CG were displayed in Fig. 5. Compared to PZ-detector, data augmentation and network architecture were found more complicated to recognize malignant lesions in CG. However, the best validation AUC of 0.94 was achieved for CG-detector when only T2-ADC pairs were used as the input. According to Fig. 1, lesions in CG are visually difficult to detect from single source of modality, which might have explained the preference of T2-ADC pair as the input. The best validation AUC for PZ-detector, on the other hand, was 0.90, when the input was a mixture type of pairs.

**Figure 6:**
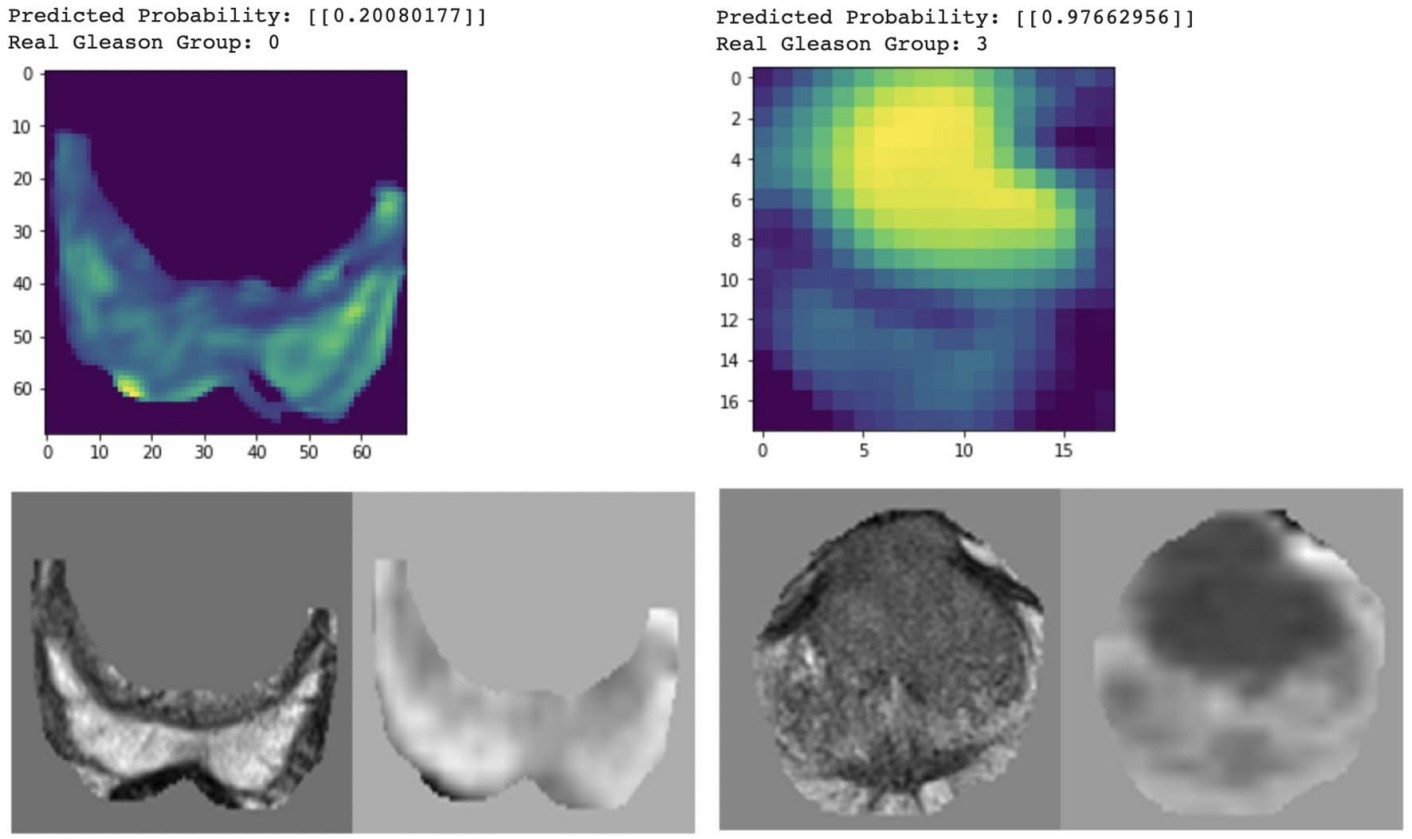
Grad-cam for PZ (left) and CG (right) respectively, showing the attention of the model aligned well with the lesion if malignant.

In Fig. 6, gradient class activation map (Grad-CAM) was used to visualize the learned PZ-detector and CG-detector. The prediction probability was the value of the output node. It can be observed from the two cases that, lesions aligned well with the hyper-intense signal in the Grad-CAM if predicted malignant, otherwise neither hyper- nor hypo-intense signal was visible.

### Challenges of domain drift

Testing data cohort was collected from our institute and posed several challenges during the inference stage.

1. Contours of PZ or CG were not available on contrary to the cases in the training cohort, but more of a realistic setting.
2. The ADC maps were calculated from DWI $b = 0$ and $b = 1000$. They are visually slightly different from the ADC maps in the training cohort.
3. Number of slices were varied in the testing cohort.

### Solutions

**Figure 7:**
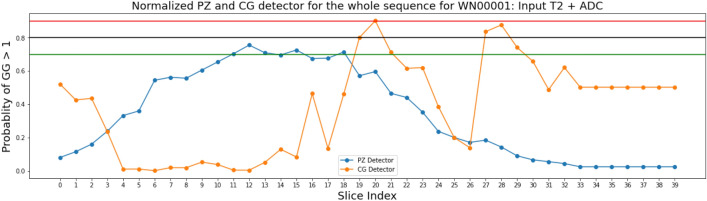
Predictions made on the sequence of Patient *WN*00001 from the independent testing cohort by PZ-detector (blue) and CG-detector (orange), respectively. Horizontal lines with red, black and green color represent the cutoff of 0.9, 0.8 and 0.7 as the probability of a lesion being malignant.
  Figure 8 was one of the successful cases where both CG-detector and PZ-detector were able to pick the suspicious slice and the Grad-CAM (Fig. 9) showed the location of suspicious lesion on that slice which aligned well with the lesion.

1. We nevertheless tested PZ-detector and CG-detector on the testing cohort, where the input is the T2-ADC pair and whole prostate gland was used as the input since sub-region contour was not available. The PZ-detector was able to accurately highlight slices with suspicious malignant lesions. While the CG-detector was able to detect lesions in CG most of the time but were found prone to the false positive traps as shown in Fig. 7, such as the marginal Slice 28, 29.

**Figure 8:**
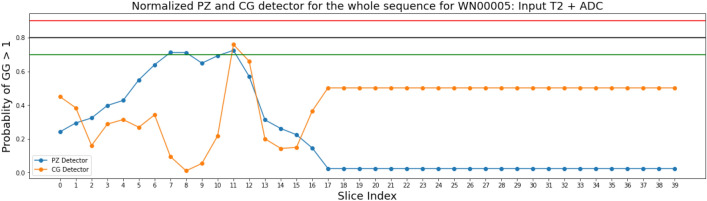
Predictions made on the sequence of Patient *WN*00005 from the independent testing cohort by PZ-detector (blue) and CG-detector (orange), respectively. Horizontal lines with red, black and green color represent the cutoff of 0.9, 0.8 and 0.7 as the probability of a lesion being malignant.

**Figure 9:**
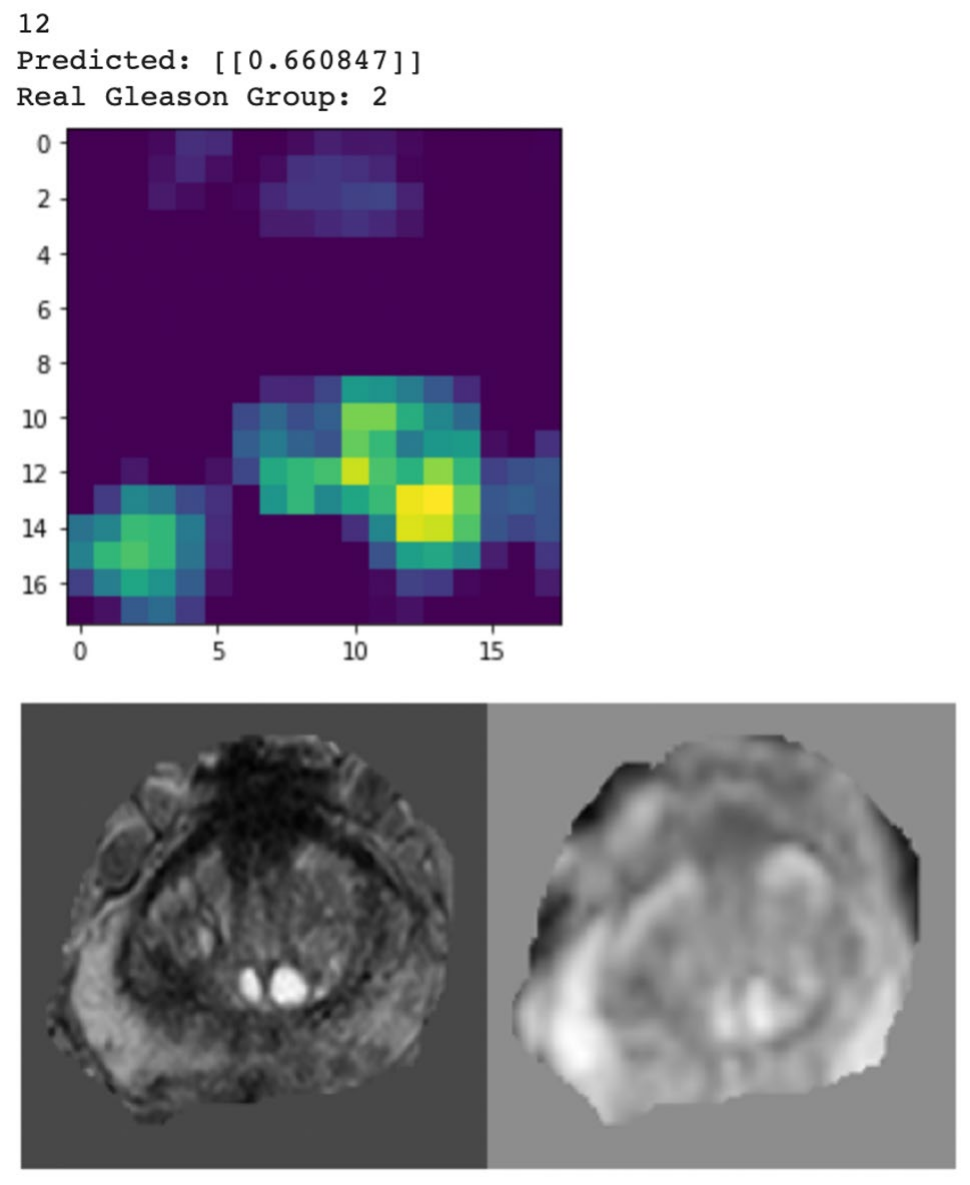
Grad-CAM from the CG-detector indicating PZ-detector and CG-detector were both sensitive to Slice 12 for Patient *WN*00005, which showed hyper-intense signal in both PZ and CG.
2. The trained models were verified robust to the domain drift issues caused by ADC map calculated from DWIs with different b values.
3. By making prediction for each slice and connecting the slices into the sequence, the PZ-detector and CG-detector were able to work together to highlight the suspicious slice out of the sequence.

## Conclusions

This work extended our previous research on using deep learning models to read from mpMRI and suggest diagnosis for lesion malignancy. The purpose was one step closer to clinical utilization by means of eliminating the manual efforts of lesion localization, utilizing automatic deep learning framework to search for the optimal augmentation strategy, network architecture and parameters, and finally make prediction for the sequence to find the most suspicious slice and localize the lesion on that slice using the saliency map. The code has been made public and ready to be deployed for anyone who is interested.

## Data Availability

The datasets used and/or analysed during the current study for testing purpose are available from the corresponding author on reasonable request.
